# Targeting chronic lymphocytic leukemia with B‐cell activating factor receptor CAR T cells

**DOI:** 10.1002/mco2.716

**Published:** 2024-09-02

**Authors:** Yaqing Qie, Martha E. Gadd, Qing Shao, Tommy To, Andrew Liu, Shuhua Li, Rocio Rivera‐Valentin, Farah Yassine, Hemant S. Murthy, Roxana Dronca, Mohamed A. Kharfan‐Dabaja, Hong Qin, Yan Luo

**Affiliations:** ^1^ Regenerative Immunotherapy and CAR‐T Translational Research Program Mayo Clinic Jacksonville Florida USA; ^2^ Department of Cancer Biology Mayo Clinic Jacksonville Florida USA; ^3^ Department of Pediatric Hematology‑Oncology University of Florida‐Jacksonville Jacksonville Florida USA; ^4^ Division of Hematology and Medical Oncology Department of Internal Medicine Mayo Clinic Jacksonville Florida USA; ^5^ Blood and Marrow Transplantation and Cellular Therapy Program Mayo Clinic Jacksonville Florida USA

**Keywords:** B‐cell malignancies, BAFF‐R, CAR T cells, chronic lymphocytic leukemia, immunotherapy

## Abstract

The challenge of disease relapsed/refractory (R/R) remains a therapeutic hurdle in chimeric antigen receptor (CAR) T‐cell therapy, especially for hematological diseases, with chronic lymphocytic leukemia (CLL) being particularly resistant to CD19 CAR T cells. Currently, there is no approved CAR T‐cell therapy for CLL patients. In this study, we aimed to address this unmet medical need by choosing the B‐cell activating factor receptor (BAFF‐R) as a promising target for CAR design against CLL. BAFF‐R is essential for B‐cell survival and is consistently expressed on CLL tumors. Our research discovered that BAFF‐R CAR T‐cell therapy exerted the cytotoxic effects on both CLL cell lines and primary B cells derived from CLL patients. In addition, the CAR T cells exhibited cytotoxicity against CD19‐knockout CLL cells that are resistant to CD19 CAR T therapy. Furthermore, we were able to generate BAFF‐R CAR T cells from small blood samples collected from CLL patients and then demonstrated the cytotoxic effects of these patient‐derived CAR T cells against autologous tumor cells. Given these promising results, BAFF‐R CAR T‐cell therapy has the potential to meet the long‐standing need for an effective treatment on CLL patients.

## Graphical abstract

BAFF‐R CAR T cells were created (A) using a lentiviral system and tested against various malignant B‐cell lines, including CLL cells. CLL patient‐derived BAFF‐R CAR T cells were also generated to test their cytotoxicity on autologous primary malignant B cells (B). The cytotoxicity of CAR T cells was measured using degranulation, interferon‐γ release, and green fluorescent protein‐labeled killing assays (C).

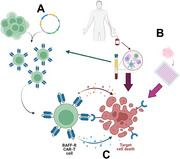

## 1 INTRODUCTION

Chronic lymphocytic leukemia (CLL) remains the most prevalent leukemia in Western countries, with 18,740 new cases reported in the United States in 2023.^1^ CLL is characterized by the accumulation of small, mature B cells in blood, bone marrow, and various lymphoid tissues; however, it is a heterogeneous malignancy with a highly variable clinical course influenced by genetic mutations that guide the clinical algorithm^2^ following specific criteria detailed in the 2018 International Workshop on CLL.^3^ Monotherapies or combination of chemoimmunotherapy that target Bcl‐2 and CD20 have transformed CLL treatment landscape.^2^, ^4^ New therapies such as ibrutinib, acalabrutinib, and venetoclax have demonstrated greater efficacy than traditional chemoimmunotherapy in CLL, especially in relapsed/refractory (R/R) setting.^5^, ^6^, ^7^ Additionally, PD‐1 immune checkpoint inhibitor has improved the overall cancer survival, particularly in the cases where CLL has transformed into Richter syndrome.^8^, ^9^ Ongoing clinical trials are exploring pembrolizumab in combination therapies.^10^, ^11^ Despite the effective targeted therapies for CLL, the primary curative option remains allogeneic hematopoietic cell transplantation (allo‐HCT).^12^, ^13^, ^14^ Unfortunately, allo‐HCT is not universally feasible due to a multitude of factors such as advanced age, existing comorbidities, and lack of a suitable donor. Moreover, the risks following hematopoietic cell allografting, including developing acute and/or chronic graft‐versus‐host disease, infections, and transplant mortality, limit success of allo‐HCT. Despite the advancement of therapeutic approaches, CLL remains largely an incurable disease.

The introduction of chimeric antigen receptors (CARs) has revolutionized hematology oncology, providing an effective treatment option for R/R B‐cell malignancies patients.^15^, ^16^, ^17^ CLL was initially targeted with CAR T‐cell therapy^18^; however, the effective application of CAR T‐cell treatment in CLL has not been fully realized even with ongoing clinical studies evaluating anti‐CD19 CAR T‐cell therapy.^19^, ^20^ In CLL, similar to R/R B‐cell malignancies, cancer cells can evade or resist therapy by losing the CD19 epitope or by suppressing CD19 expression due to genetic alterations such as splice variation, mutations, or lineage switching.^21^, ^22^, ^23^ Addressing the unmet medical need for patients facing relapse remains a critical challenge.

To fulfill the demand for CLL‐specific CAR T‐cell therapy, efforts are focused on identifying robust targets that are essential for B‐cell survival. One promising target is the B‐cell activating factor receptor (BAFF‐R), a specific marker for B‐lymphocyte development and mature B‐cell survival.^24^, ^25^ BAFF‐R is expressed on the mature B cells^26^, ^27^; however, its expression is also observed in pre‐B cells that show activation within the promoter of the *TNFRSF13C* gene that encodes BAFF‐R.^28^, ^29^ BAFF‐R is particularly relevant in CLL due to its high expression in clonally expanded mature B cells within this disease context.^26^, ^30^ The BAFF/BAFF‐R cascade provides a survival signal that malignant B cells in CLL exploit to evade apoptosis, a process further sustained by the CLL microenvironment.^31^, ^32^ The extensive expression of BAFF‐R in B‐cell malignancies, along with its crucial role in tumor cell survival, makes BAFF‐R an appealing target for CAR T‐cell therapy in CLL.^33^

BAFF‐R CAR T cells have been methodically engineered and characterized in various in vitro assays, specifically exhibiting cytotoxic efficacy in xenograft models of R/R acute lymphoblastic leukemia (R/R ALL) with CD19 antigen loss.^34^, ^35^ This has led to a phase 1 clinical trial for BAFF‐R CAR T cells in Non‐Hodgkin lymphoma (NHL) (NCT05370430). In the study presented here, we hypothesized that BAFF‐R CAR T‐cell therapy would be well‐suited for CLL. We confirmed that we are able to generate BAFF‐R CAR T cells, which consistently met established quality control (QC) standards. Most significantly, we showed the efficacy of these BAFF‐R CAR T cells against three CLL cell lines, CD19‐knockout (KO) CLL cells, and primary tumor cells from CLL patients. Furthermore, we developed BAFF‐R CAR T cells from CLL patients and demonstrated their cytotoxicity against CLL tumor cells in an autologous context.

## 2 RESULTS

### 2.1 Verification of antigen‑specific functionality of BAFF‐R CAR T cells

**FIGURE 1:**
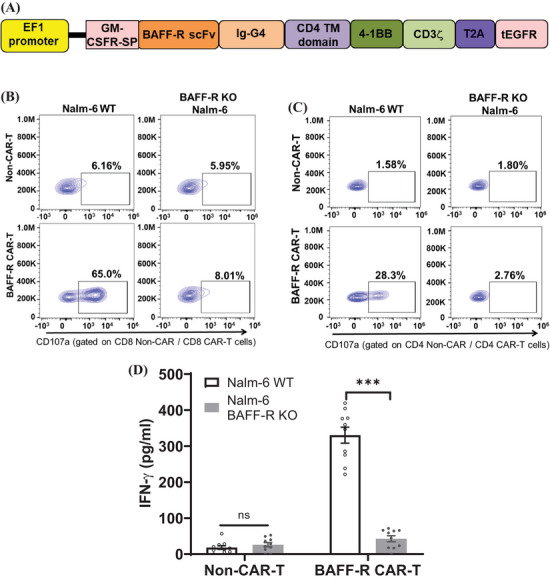
Verification of antigen‑specific functionality of B‐cell activating factor receptor (BAFF‐R) chimeric antigen receptor (CAR) T cells. (A) This schematic diagram depicts BAFF‐R scFv and additional domains of the CAR engineered into the lentiviral expression vector. (B and C) Antigen‐specific cytotoxicity of BAFF‐R CAR T cells were evaluated by a CD107a assay with representative data shown here. CAR T cells were co‐incubated with target cells (WT or BAFF‐R‐knockout [KO] Nalm‐6 cells) at an E:T ratio of 2:1. Cytotoxic analysis was focused on CD8^+^ CAR T cells (B) or CD4^+^ CAR T cells (C); non‐CAR T served as control samples. The BAFF‐R CAR T cells that were generated from three donors and when incubated with target cells showed similar cytotoxicities (Table S2). (D) Interferon‐gamma (IFN‐γ) was measured from the harvested supernatant of BAFF‐R CAR T cells that were co‐cultured with target cells (WT or BAFF‐R‐KO Nalm‐6 cells) at an E:T ratio of 4:1 for 72 h. The results were presented as mean ± SEM from four replicates and representative from three independent experiments (^∗∗∗^ *p* < 0.001; ns, no significant differences).

We developed a second‐generation BAFF‐R CAR using a clinical vector, as previously reported (Figure 1A).^34^ The manufacturing processes in our platform have consistently produced high‐quality BAFF‐R CAR T cells, yielding reproducible batches that met established QC standards. These criteria included assessments of cell quality, CAR T characterization, and safety measurements (Table S1).

**FIGURE 2:**
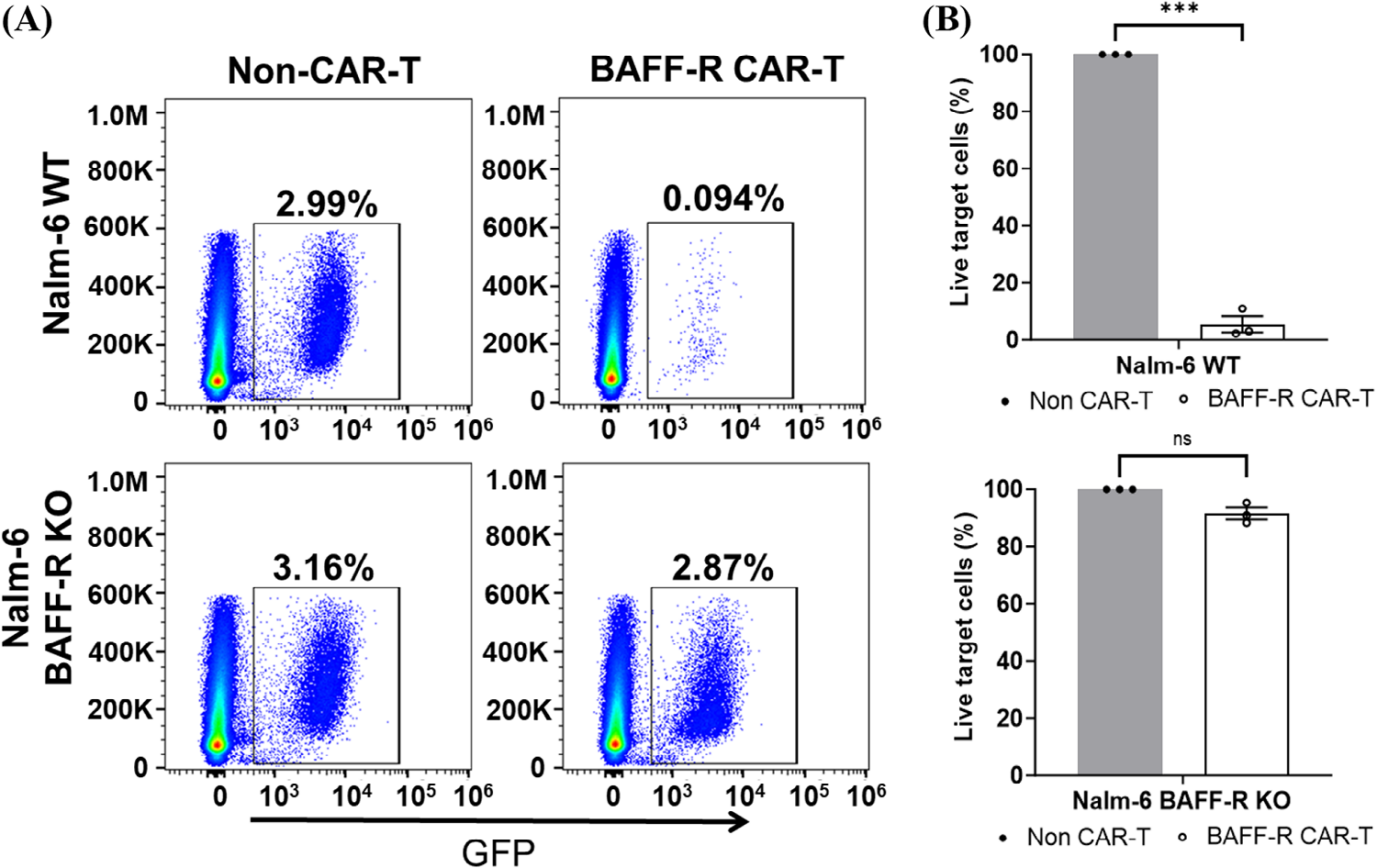
Cytotoxicity of B‐cell activating factor receptor (BAFF‐R) chimeric antigen receptor (CAR) T cells on acute lymphocytic leukemia (ALL) cells directly confirms cytolysis of target cells. (A) Using a direct killing assay, BAFF‐R CAR T cells or non‐CAR T cells were co‐cultured with either green fluorescent protein (GFP)‐labeled tumor cells (Nalm‐6 WT or BAFF‐R‐knockout [KO] cells) at 20:1 (E:T ratio) for 24 h. The live GFP‐expressing tumor cells were quantified with a gating strategy that gated on the live cells first and then the GFP‐positive target cells. These dot plots are representative of data obtained from three independent experiments. (B) By setting the non‐CAR T cells at 100% for each of three experiments, the non‐CAR T group served as a baseline to normalize the percentile of live tumor cells in BAFF‐R CAR T group. The results were showed as mean ± SEM from four replicates and representative from three independent experiments (^∗∗∗^ *p* < 0.001).

The antigen‐specific cytotoxicity of BAFF‐R CAR T cells was evaluated using BAFF‐R‐positive (WT) and BAFF‐R‐negative (BAFF‐R‐KO) Nalm‐6 cells. As a control, non‐CAR T cells were used. Our study demonstrated that both CD4**^+^** and CD8**^+^** CAR T cells showed antigen‐specific activation against Nalm‐6 Wild type (WT), indicated by an increased CD107a expression. The lack of T cells activation against BAFF‐R‐KO Nalm‐6 confirmed the antigen‐specific functionality of the BAFF‐R CAR T cells (Figure 1B for CD8^+^ and Figure 1C for CD4^+^; Table S2 collates the degranulation results of three separate experiment). BAFF‐R CAR T exhibited a significant release of interferon‐gamma (IFN‐γ) when incubated with Nalm‐6 WT, but not with BAFF‐R‐KO Nalm‐6 cells (Figure 1D), which was further confirmed antigen specific cytotoxicity. The direct cytolysis of tumor cells by BAFF‐R CAR T cells was assessed with green fluorescent protein (GFP)‐labeled Nalm‐6 WT cells. The GFP‐labeled Nalm‐6 WT cells were co‐cultured with BAFF‐R CAR T cells for 24 h; then, followed the evaluation by flow cytometry and gated on alive GFP‐positive cells (Figure 2A). The large excess of CAR T cells skews the data to this large T‐cell population; therefore, the data were evaluated and plotted to focus on the changes in the percentage of alive tumor cells. When normalized to the non‐CAR T‐cell sample in which no antigen specific killing is expected, a significant reduction in GFP‐labeled Nalm‐6 WT cells when incubated with BAFF‐R CAR T cells was clearly resolved (Figure 2B).

### 2.2 In vitro cytotoxicity of BAFF‐R CAR T cells against CLL cell lines

**FIGURE 3:**
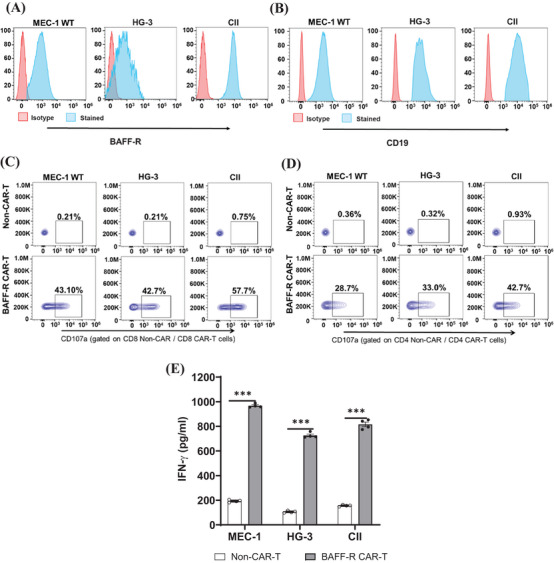
Cytotoxicity of B‐cell activating factor receptor (BAFF‐R) chimeric antigen receptor (CAR) T cells against three chronic lymphocytic leukemia (CLL) cell lines in vitro. BAFF‐R (A) or CD19 (B) expression in MEC‐1 WT, HG‐3, or CII cell lines were characterized by BAFF‐R‐AF647 or CD19 APC antibodies staining and assessed using flow cytometry. (C and D) BAFF‐R CAR T cells were incubated with MEC‐1 WT, HG‐3, or CII cells at an E:T ratio of 2:1. CD107a‐positive cell analysis, which was utilized to indicate antigen‐specific cytotoxicity, was focused on CD8^+^ (C) or CD4^+^ CAR T cells (D); non‐CAR T cells were used as negative controls. (E) Additionally, cytotoxicity of BAFF‐R CAR T cells was observed with the co‐incubated with either MEC‐1 WT, HG‐3, or CII cell lines at an E:T ratio of 4:1. Interferon‐gamma (IFN‐γ) was measured from the harvested supernatant after 72 h. Non‐CAR T cells were used as controls. The results were showed as mean ± SEM from four replicates and representative from three independent experiments (^∗∗∗^ *p* < 0.001).

After confirming the antigen‐specific cytotoxicity of BAFF‐R CAR T cells, we further evaluated the cytotoxicity of the CAR T cells against three CLL cell lines: MEC‐1, HG‐3, and CII. BAFF‐R/CD19 expression was validated on these cell lines (Figure 3A,B). By gating on the CAR marker EGFR, we determined the percentage of CD107a‐positive cells in both CD8**^+^** CAR T cells (Figure 3C) and CD4**^+^** CAR T cells (Figure 3D). Significantly increased percentages of activated CD8^+^ CAR T cells and CD4^+^ CAR T cells are observed after coincubation with these three CLL cell lines compared to the non‐CAR T groups. As expected, higher activation was observed in CD8**^+^** CAR T cells, as previously described.^36^ We also confirmed the cytotoxicity of BAFF‐R CAR T cells against these three CLL cell lines by the measured significant release of IFN‐γ, compared to the non‐CAR T group (Figure 3E).

### 2.3 BAFF‐R CAR T cells retained their cytotoxicity against CD19‐KO CLL cells

**FIGURE 4:**
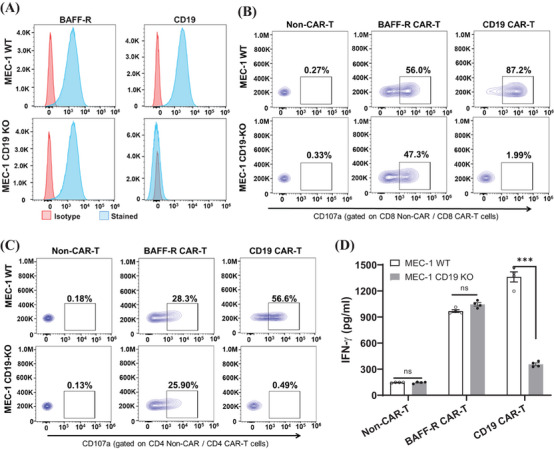
Cytotoxicity of B‐cell activating factor receptor (BAFF‐R) chimeric antigen receptor (CAR) T cells against CD19‐knockout (KO) MEC‐1 cell line. (A) BAFF‐R or CD19 expression in MEC‐1 WT or MEC‐1 CD19‐KO were characterized by BAFF‐R‐AF647 or CD19 APC antibodies staining and analyzed with flow cytometry. (B and C) BAFF‐R or CD19 CAR T cells were incubated with MEC‐1 WT or MEC‐1 CD19‐KO at an E:T ratio of 2:1, respectively. Cell surface expression of CD107a was analyzed on gated CD8^+^ CAR T cells (B) or CD4^+^ CAR T cells (C); non‐CAR T cells were used as controls. (D) BAFF‐R or CD19 CAR T cells were co‐cultured with either MEC‐1 WT or MEC‐1 CD19‐KO cell lines at an E:T ratio of 4:1. IFN‐γ was assessed from the harvested supernatant after 72 h. Non‐CAR T cells were used as control. The results were showed as mean ± SEM from four replicates and representative from three independent experiments (^∗∗∗^ *p* ****< 0.001; ns, no significant differences).

Given the growing reports of antigen‐escape relapse in patients treated with CD19 CAR T cells, our next experiment was the functional characterization of BAFF‐R CAR T cells against CD19‐KO CLL. For this purpose, we utilized CD19‐KO MEC‐1 (MEC‐1 CD19‐KO) cells as a representative cell line for CD19 antigen loss. We validated the presence of BAFF‐R in both MEC‐1 WT and MEC‐1 CD19‐KO cell lines and confirmed the absence of CD19 in the MEC‐1 CD19‐KO cells (Figure 4A). BAFF‐R CAR T and CD19 CAR T cells were generated from the same donor, respectively. The potency of these two CAR T cells was comparable, and non‐CAR T cells were set as a negative control (Figure S1A). The cytotoxic efficacy of BAFF‐R CAR T against CD19‐KO MEC‐1 was assessed by a CD107a assay, using non‐CAR T cells and CD19 CAR T cells as controls. The activated CD8**^+^** CAR T cells (Figure 4B) and CD4**^+^** CAR T cells (Figure 4C) following incubation with either MEC‐1 WT or MEC‐1 CD19‐KO cell lines showed antigen‐specific cytotoxicity. The activated BAFF‐R CAR T cells was not dependent upon CD19 expression on target cells; however, activated CD19 CAR T cells were observed only against MEC‐1 WT and not the MEC‐1 CD19‐KO cells (Figure 4B,C; direct killing assay in Figure S1B). The BAFF‐R CAR T cells and target cells were further evaluated in the GFP direct killing assay and confirmed cytolysis of MEC‐1 and MEC‐1 CD19‐KO cells by BAFF‐R CAR T cells (Figure S2A‒D). The cytotoxicity of BAFF‐R CAR T against CD19‐KO cells was further confirmed by the significantly elevated release of IFN‐γ upon interaction with both MEC‐1 CD19‐KO and MEC‐1 WT cell lines. In contrast, CD19 CAR T cells exhibited an increase released level of IFN‐γ only in response to MEC‐1 WT (Figure 4D).

### 2.4 BAFF‐R CAR T cells exhibited cytotoxicity against primary tumor cells obtained from CLL patients

**FIGURE 5:**
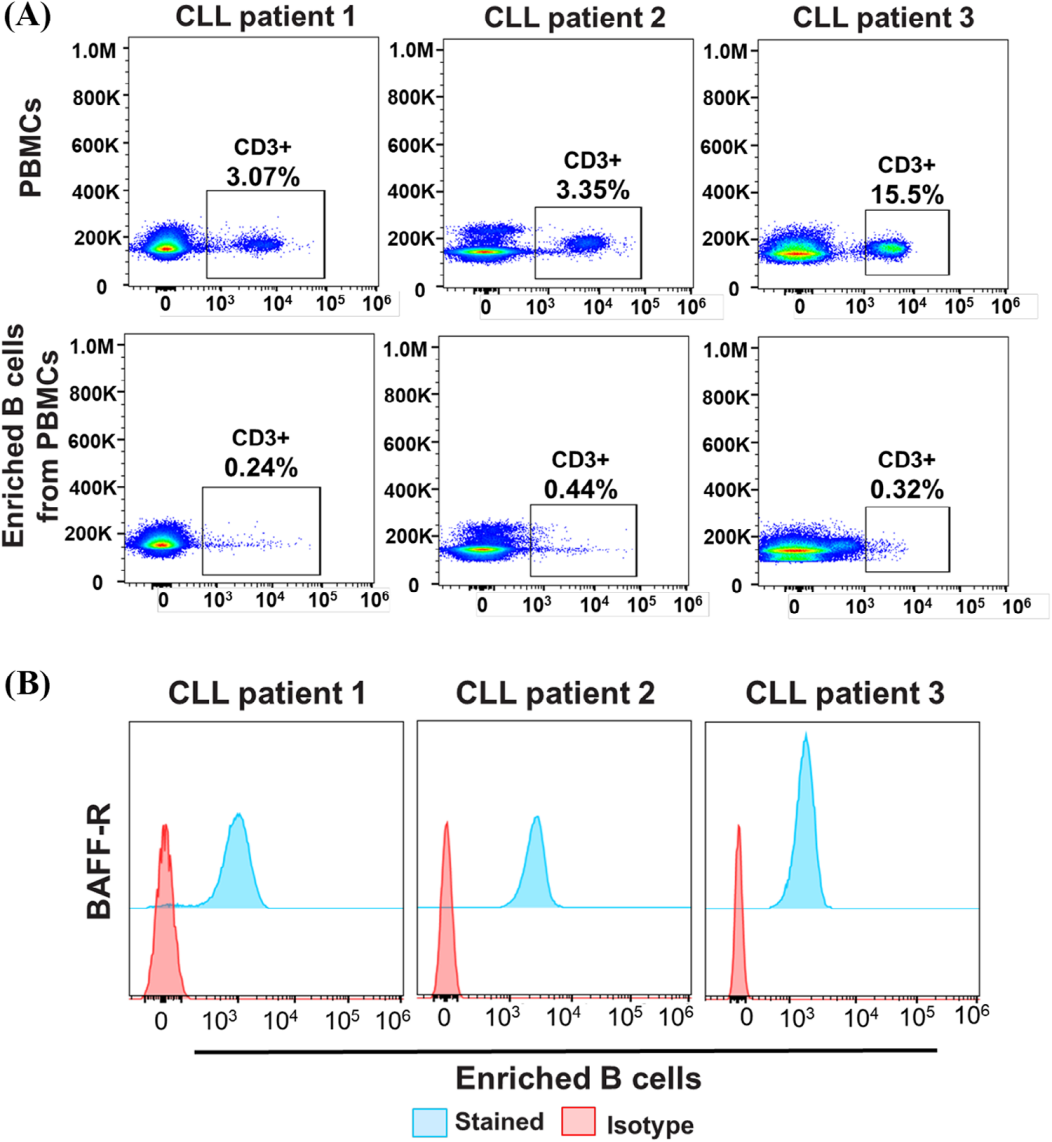
Immunophenotyping of isolated primary B tumor cells from chronic lymphocytic leukemia (CLL) patients. (A) Primary B tumor cells from CLL patients were isolated from peripheral blood mononuclear cells (PBMCs) to serve as target cells in chimeric antigen receptor (CAR) T‐cell function assays. Enriching B cells significantly reduces the proportion of T cells in the target cell population. (B) B‐cell activating factor receptor (BAFF‐R) expression in the enriched B cells from the three CLL patients was characterized using BAFF‐R‐AF647 antibodies, followed by flow cytometry analysis. Isotype antibody served as staining controls.

**FIGURE 6:**
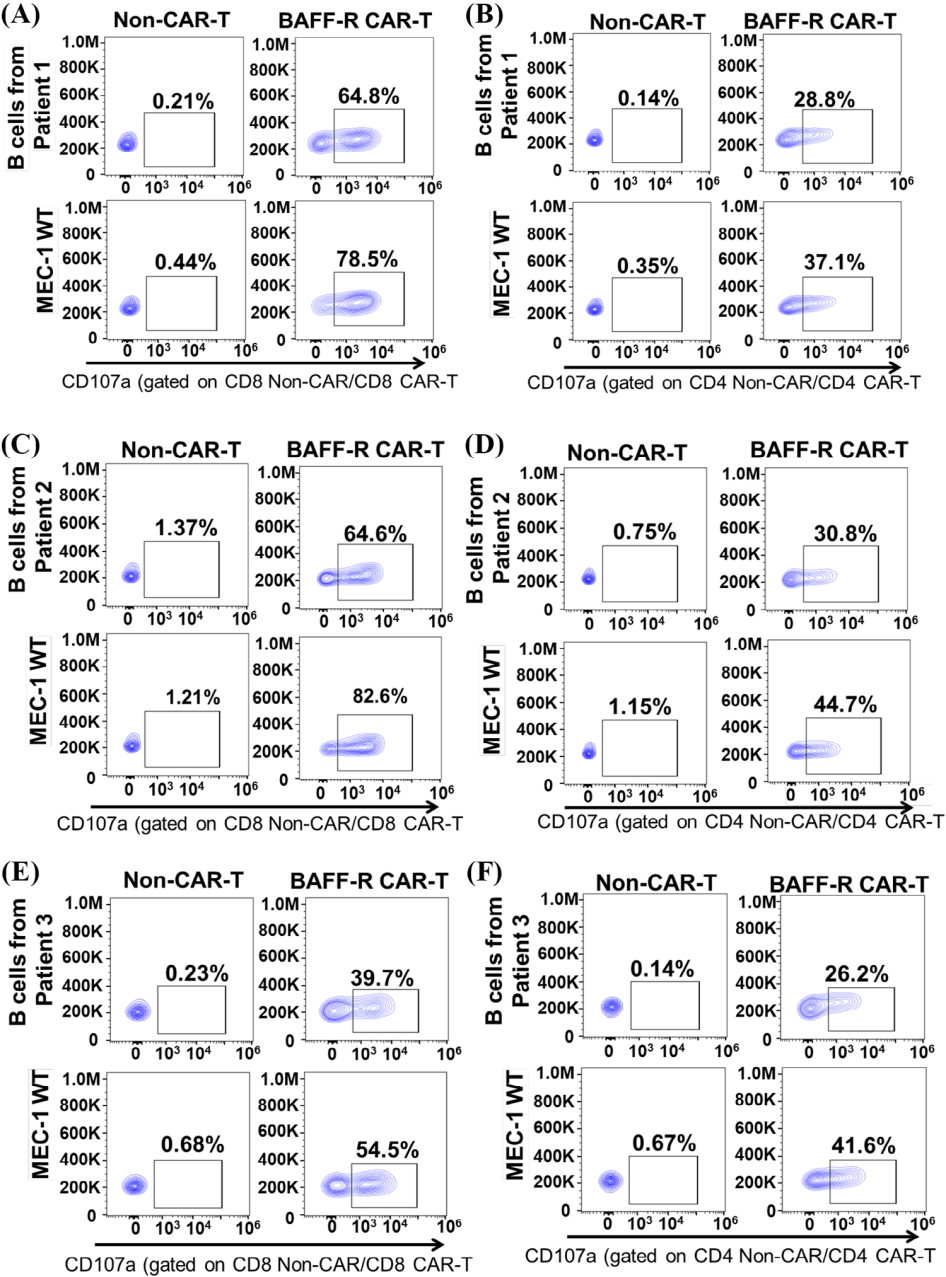
Cytotoxicity of B‐cell activating factor receptor (BAFF‐R) chimeric antigen receptor (CAR) T cells from healthy donors against primary B tumor cells from chronic lymphocytic leukemia (CLL) patients. The BAFF‐R CAR T cells from healthy donors were produced and then incubated with primary B tumor cells from three CLL patients at 2:1 ratio, followed by a CD107a assay. Used MEC‐1 WT cells as a positive control. The analysis focused on CD8^+^ or CD4^+^ CAR T cells, with non‐CAR T cells as negative controls. Cytotoxicity of BAFF‐R CAR T cells derived from healthy donor A against primary B tumor cells from three CLL patients was observed: CLL patient 1 (CD8^+^ in A; CD4^+^ in B); CLL patient 2 (CD8^+^ in C; CD4^+^ in D); and CLL patient 3 (CD8^+^ in E; CD4^+^ in F).

Having confirmed the cytotoxicity of BAFF‐R CAR T cells against CLL cell lines, we next evaluated their cytotoxicity against primary tumor cells obtained from CLL patients. Patient B cells were enriched from the peripheral blood mononuclear cells (PBMCs) of three CLL patients (Figure 5A and Table S3). The positive expression of BAFF‐R on B cells was validated on all the patients (Figure 5B), supporting BAFF‐R CAR T therapy as an ideal therapy to target CLL. Three batches of BAFF‐R CAR T cells were generated from healthy donors. In a cytotoxic degranulation assay, these batches of CAR T cells were consistently be activated when targeting on primary B cells isolated from CLL patient, as expected. Conversely, no significant activity was showed in non‐CAR T group (Figure 6).

### 2.5 CLL patient‐derived BAFF‐R CAR T cells specifically targeted autologous primary B tumor cells

**FIGURE 7:**
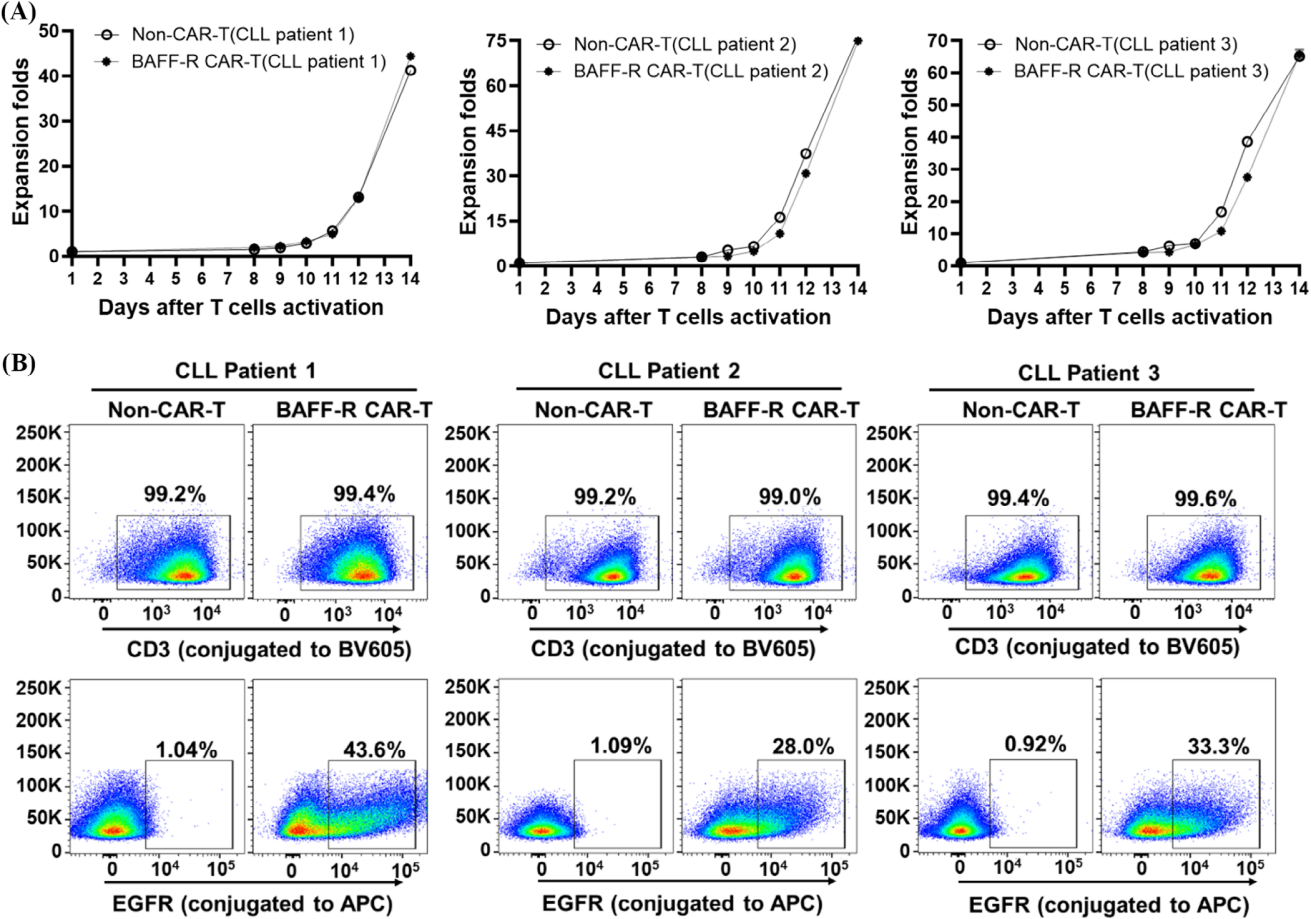
Generation of B‐cell activating factor receptor (BAFF‐R) chimeric antigen receptor (CAR) T cells derived from chronic lymphocytic leukemia (CLL) patients. (A) The growth curves of BAFF‐R CAR T and their matched non‐CAR T cells, obtained from T cells of three CLL patients, were displayed side by side for comparison. (B) The identity (CD3‐positive staining) greater than 80% and potency (tEGFR staining) greater than 10% were detected in the patient‐derived CAR T cells.

**FIGURE 8:**
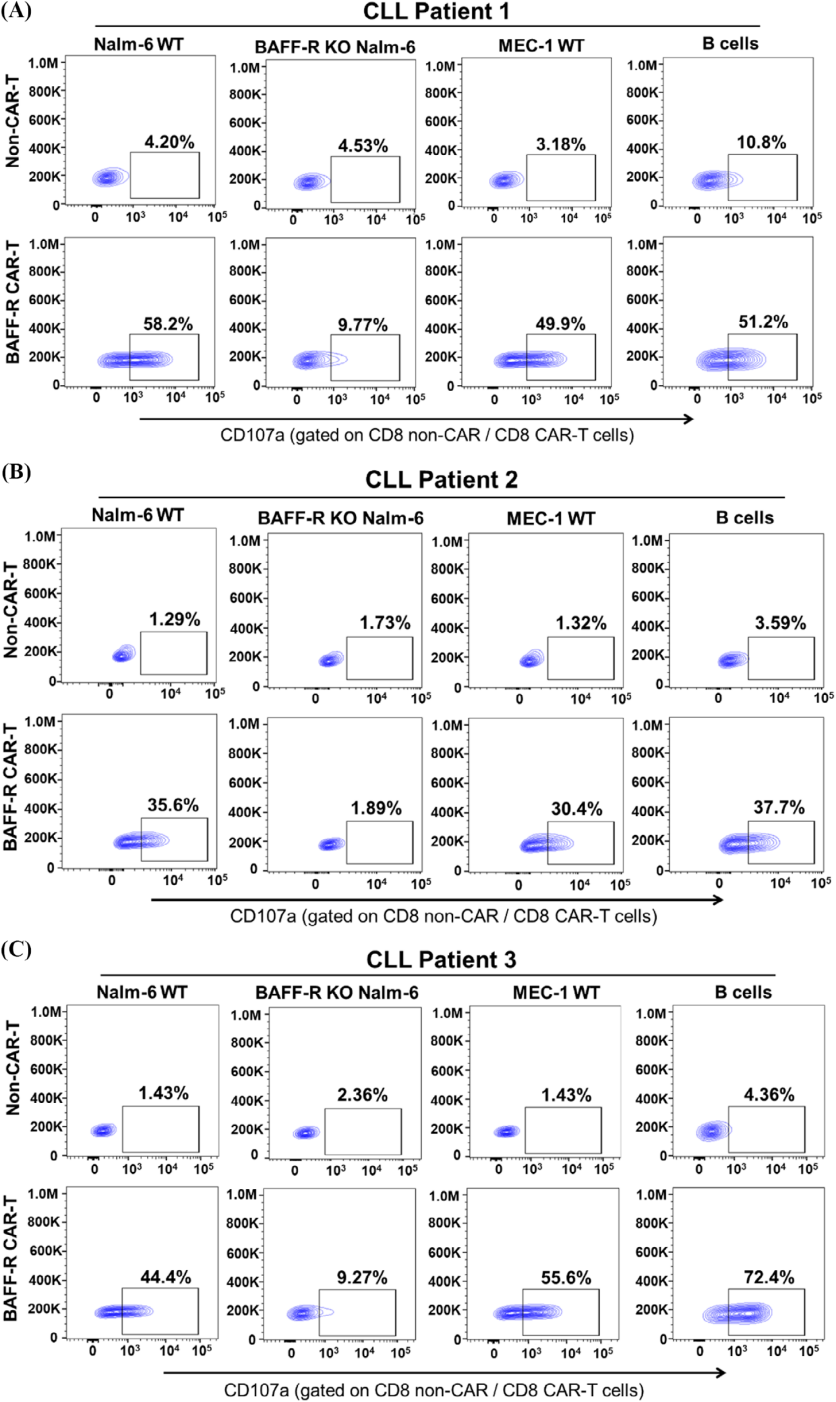
Chronic lymphocytic leukemia (CLL) patient‐derived B‐cell activating factor receptor (BAFF‐R) chimeric antigen receptor (CAR) T elicited ex vivo cytotoxicity against autologous B cells. Using a CD107a degranulation assay, the cytotoxic efficacy of CLL patient‐derived BAFF‐R CAR T cells was assessed against a collection of previously established target cells: Nalm‐6 WT, BAFF‐R‐KO Nalm‐6, and MEC‐1 WT cells. Most significantly, the cytotoxic efficacy of CLL patient‐derived BAFF‐R CAR T cells showed cytotoxicity against autologous B tumor cells. Non‐CAR T cells from the same patient were used as a control. Cytotoxicity analysis of was performed on the CD8^+^ CAR T‐cell populations from CLL patient 1 (A), CLL patient 2 (B), and CLL patient 3 (C). Note that the cytotoxicity was also gated on the CD4^+^ CAR T‐cell population and included in Figure S2A‒C.

So far, the functionality of BAFF‐R CAR T cells derived from healthy donors on CLL tumors have been confirmed. Acknowledging the potentially compromised T‐cell function within CLL patients, we conducted a study to assess the cytotoxicity of CAR T cells derived from CLL patients against their own primary tumor cells, highlighting the translational significance of BAFF‐R CAR T cells. In accordance with our laboratory standard operating procedures for CAR T‐cell manufacture, we have made three batches of BAFF‐R CAR T cells from CLL patients. Each batch of these CAR T cells achieved QC standards, which is over 25× expansion folds (Figure 7A) and meeting the required characteristics: identity (CD3‐positive staining) greater than 80% and potency (tEGFR staining) greater than 10% (Figure 7B). To characterize CAR T function, we first validated whether the CAR T cells derived from patients could be activated by specific antigens. This was demonstrated by increased CD107a expression in a degranulation assay when the CAR T cells were exposed to Nalm‐6 WT cells, but not to BAFF‐R‐KO Nalm‐6 target cells (Figure 8A‒C, the left two panels). Most significantly, the cytotoxicity of the three patients’ BAFF‐R CAR T cells against their own primary B tumor cells was readily observed when gated on CD8**^+^** CAR T (Figure 8A‒C, the right two panels). We also observed cytotoxicity of these CAR T cells from patients against MEC‐1 WT cells. We noticed T‐cell activation background in one patient (patient 1), which may reflect the baseline of anti‐tumor immunity in this specific patient.^33^ Direct killing, as determined by the loss of GFP‐labeled target cells, also confirmed the cytolytic activity of patient derived BAFF‐R CAR T cells (patient 2) against MEC‐1 WT and MEC‐1 CD19‐KO cell lines (Figure S2E,F).

## 3 DISCUSSION

The application of CAR T‐cell therapies for various B‐cell malignancies has positioned CAR T cells as an impressive treatment option. CD19 CAR T‐cell therapy becomes a treatment option when patients relapse or become refractory to conventional therapies. However, the use of FDA‐approved CD19 CAR T cells for B‐cell malignancies has demonstrated a decrease in overall survival (OS) after 5 years. Specifically, the OS was 42.6% for patients with refractory large B‐cell lymphoma who received an infusion of axicabtagene ciloleucel.^37^ CLL patients experience considerably lower complete remission rates with CD19 CAR T‐cell treament.^38^, ^39^ The CAR T‐cell therapy may be ineffective when the cancer cells change the quantity or the actual surface CD19 epitope expression through one or several genetic mechanisms such as splice variation, mutations, or lineage switching,^21^, ^22^, ^23^ thus allowing the cancer cells to escape recognition by the scFv of the CAR T cells. Suboptimal CD19 CAR T cells cytotoxicity may be observed even when CD19 surface expression is maintained on cancer cells. Past therapeutic exposure, intrinsic T‐cell deficiencies in the CLL patient population (advanced age and comorbidities), and the immunosuppressive microenvironment of CLL may contribute to an ineffectual CAR T‐cell product, causing a higher rate of non‐response or relapse in CLL patients.^21^, ^40^, ^41^ This unmet medical need for CLL patients experiencing relapse remains.

R/R B‐cell malignancies after a single‐antigen CAR T‐cell treatment emphasizes the need for alternative treatment approaches. Strategies such as pre‐treatment with ibrutinib before CD19 CAR T‐cell treatment have been explored.^42^, ^43^ Another approach involves identifying new target antigen for novel CAR design. CD19, chosen for its expression throughout the B‐cell life cycle and critical role in B‐cell receptor signal transduction, has been a primary focus.^44^ For addressing B‐cell pathologies and malignancies, BAFF and its receptor (BAFF‐R) have emerged as prospective targets due to their crucial role in B‐cell survival.^25^, ^26^ While BAFF monoclonal antibodies are being investigated for autoimmune diseases,^45^ their initial standalone efficacy against B‐cell malignancies has been limited.^24^, ^46^ However, there is ongoing exploration of combining BAFF monoclonal antibodies with ibrutinib in a CLL clinical trial (NCT03400176). We have chosen to focus on the other key partner in this cascade: BAFF‐R. Both BAFF‐R monoclonal antibody and BAFF‐R CAR T cells treatments have shown cytotoxic efficacy against various B‐cell malignancies, including acute lymphocytic lymphoma, follicular lymphoma, mantle cell lymphoma, and diffuse large B‐cell lymphoma.^35^, ^47^ Within R/R B‐cell malignancies, the critical role of BAFF‐R in the survival of mature B cells makes loss of BAFF‐R and subsequent antigen escape unlikely, or if BAFF‐R were lost, malignant B‐cell proliferation would be significantly impaired. We and others show BAFF‐R‐positive expression on CLL primary tumor cells (Figures 5B and S3).^26^, ^27^, ^33^, ^48^, ^49^ The anti‐tumor efficacy of BAFF‐R CAR T cells has been observed in both ex vivo primary tumor co‐incubation assays and in vivo xenograft models using some of these same cell lines, leading to the initiation of a phase I clinical trial for R/R ALL patients (NCT04690595).^34^, ^35^ Encouraged by the activity observed with BAFF antibodies and the promising preliminary results of BAFF‐R CAR T treatment against B‐cell malignancies in the previous study, we have designed a series of experiments focusing on BAFF‐R CAR T therapy as a possible therapeutic approach for CLL patients.

To ensure a reproducible product for our research needs and to facilitate future translational clinical applications, we standardized our CAR T‐cell manufacturing process. After confirming the positive expression of BAFF‐R on three CLL cell lines, we assessed the antigen‐specific cytotoxicity of our BAFF‐R CAR T cells using CD107a degranulation and cytokine release assays. We observed comparable T‐cell activity in BAFF‐R CAR T groups against these three cell lines. CD19 CAR T‐cell therapy has shown suboptimal results in CLL clinical trials and has raised concerns of CD19‐negative escape in CLL and other B‐cell malignancies. To address this, we engineered a CD19‐KO CLL cell line to mimic clinical CD19 antigen loss. Our findings indicate retained in vitro cytotoxic efficacy by BAFF‐R CAR T cells targeting on CD19‐KO CLL cells. The anti‐tumor efficacy of BAFF‐R CAR T therapy in NOD scid gamma mouse (NSG) mouse models using ALL and NHL cell lines have been confirmed^35^, ^47^; unfortunately, we experienced challenges in engineering HG3 and CII cell lines to robustly express luciferase. MEC‐1 proved a valuable in vitro model; however, when applied to an in vivo model with NSG mice, we observed unexpected high non‐CAR T‐cell alloreactivity but no adverse events due to CAR T‐cell treatment. Challenges with CLL cell line may be attributed to the Epstein‒Barr virus seropositivity of CLL cell lines that does not favor engraftment into immunosuppressed mice.^50^ The Eμ‐TCL1 transgenic mouse model exhibits many of the immunological dysregulation and dysfunction observed in CLL^51^; but immunocompetent mice are not acceptable models for testing human CAR T cells. The Eμ‐TCL1 model does support the growing view that the microenvironment (e.g., chemokine receptors in lymphoid tissue^52^ and stimulating antigens^53^) strongly affects CLL cells.^32^ Therefore, we turned our attention to using CLL patients primary tumor cells as our targets for BAFF‐R CAR T cells.

Acknowledging the significant challenges posed by CAR T manufacturing from blood cancer patients that includes low T‐cell quantity and impaired T‐cell function, we designed a proof‐of‐concept study that would isolate both T cells and B cells from CLL patient blood samples. We first confirmed BAFF‐R expression on B cells from CLL patient and then successfully generated CLL patients derived CAR T cells that were functionally tested against both BAFF‐R‐positive cell lines and autologous primary tumor cells. The cytotoxicity of three patient‐derived CAR T cells against autologous primary B cells confirms our hypothesis that BAFF‐R CAR T cells treatment holds a promise as an effective therapy for CLL patients. These results highlight the feasibility and anti‐tumor efficacy of CLL patient‐derived CAR T cells in a clinical context. We are also encouraged by the recent positive safety report of BAFF‐R CAR T cells with low and reversible Cytokine release syndrome (CRS) and Immune effector cell‐associated neurotoxicity syndrome (ICANS) in three mantle cell lymphoma patients.^54^ BAFF‐R CAR T treatment provides an additional tool to the clinical armamentarium, especially patients with reduced CD19 responsiveness.

We acknowledge that this study has limitations due to the small sample size of patients that were used to isolate cellular fractions and that only eight CLL were included in this proof‐of‐concept study. Any comparisons between differences in expansion or activity of the final product are premature and more suitable for correlative studies that accompany a clinical trial or would require a more advanced analysis of transcriptomic changes between patient final CAR T‐cell products. Although traditional in CAR T‐cell studies, including in vivo testing is standard; however, as discussed, CLL in vivo models may not be predictive which led to our decision to develop ex vivo CLL primary cell models. The modulatory effect of the tumor microenvironment is evident in CLL with the functional success of BTKi inhibitors and when comparing peripheral blood CLL cells to active CLL in lymphoid tissue.^55^, ^56^ Developing models to mimic the tumor microenvironment in CLL would be beneficial to improving our understanding of CLL and, by extension, guide our exploration of therapies.

In the context of CLL treatment, researchers and clinicians are exploring other antigen‐specific CAR T cells, such as CD22 CAR T‐cell therapy, which has advanced into CLL patients clinical trials with promising results and tolerability.^20^, ^57^ Despite the success of single‐target CAR T cells, evidence suggests an increased risk of relapse as post‐treatment time increases. The challenge of harvesting sufficient T cells from older patients with R/R B‐cell malignancies further complicates the success of a second CAR T‐cell infusion. To combat these challenges, a dual‐targeting, bispecific CD19/BAFF‐R CAR T‐cell platform has been explored, addressing the antigen heterogeneity observed in ALL.^58^ Other strategies, such as dual CD19/CD22 CAR T cells and sequential administration of CD19 and CD22 CAR T cells, are under active investigation.^59^, ^60^

The performance of CD19 CAR T‐cell therapy against CLL has been challenging compared to other B‐cell malignancies.^61^ The U.S. Food and Drug Administration recently approved lisocabtagene maraleucel (liso‐cel) for the treatment of CLL and small lymphocytic lymphoma (SLL); liso‐cel, a CD19 targeted CAR T‐cell therapy, is administered as equal doses of CD8 and CD4 CAR T cells.^62^ Approval followed the favorable findings of the TRANSCEND CLL 004 on‐going trial that evaluated R/R CLL patients who have had two lines of therapy, including BTK inhibitor, who received 100 × 10^6^ CAR T cells and had an objective response rate of 43% and total median OS of 30.3 months.^62^ This increased dose requirement may still be a challenge for some CLL patients and relapse may still occur due to factors such as T‐cell exhaustion, antigen loss, and T‐cell dysfunction within the immunosuppressive lymphoid environment.^32^ In this study, we have addressed the challenge of antigen escape/loss with the introduction of BAFF‐R as a targetable antigen with the goal of positive clinical trials results. Indeed, first clinical trials will also potentially include patients who have relapsed after single CD19 CAR T‐cell therapy. With a new actionable target, we will begin to amour BAFF‐R CAR T cells with supportive molecules that can either ameliorate T‐cell exhaustion or combat the immunosuppressive tumor microenvironment associated with CLL. This challenge necessitates the consideration of adjuvant therapies or CAR designs that incorporate elements to enhance or support intrinsic T‐cell functions. The correlative studies that are comparing CD19 CAR T‐cell responders to non‐responders are significantly contributing to the understanding of the genes and proteins in a successful CD19 CAR T cell.^61^, ^63^ These studies highlight critical pathways that can be targeted by elements engineered into future CARs to improve in vivo functions of the next generation of CAR T cells.

## 4 MATERIALS AND METHODS

### 4.1 Cell lines

Nalm‐6, MEC‐1, HG‐3, and CII cell lines were purchased from Deutsche Sammlung von Mikroorganismen und Zellkulturen GmbH (DSMZ, Braunschweig). Cells were cultured with either 90% RPMI 1640 or Iscove's MDM (Thermo Fisher) contained with 10% heat‐inactivated fetal bovine serum (HI‐FBS) (Thermo Fisher). Jurkat and 293FT cell lines were bought from American Type Culture Collection and cultured with either 90% Dulbecco's modified Eagle medium or RPMI 1640 (Thermo Fisher) containing 10% HI‐FBS. Prior to expansion, usage, and cryopreservation, cell lines were authenticated by flow cytometry by staining with anti‐BAFF‐R‐AF647 and anti‐CD19‐PE (both from BD Bioscience). A BAFF‐R‐KO in Nalm‐6 (BAFF‐R‐KO Nalm‐6), GFP expressing Nalm‐6 cell lines, and a representative CD19‐deficient MEC‐1 cell line (MEC‐1 CD19‐KO) were previously generated.^33^ Routine mycoplasma contamination testing was performed on all cell lines.

### 4.2 Isolate PBMCs and Tn/mem cells from the blood of healthy donors

Isolating PBMCs and naïve/memory T cells (Tn/mem) from healthy donors follows the procedures described previously.^33^, ^35^, ^47^, ^64^ In briefly, using leukocyte reduction system cones, as conducted by the Division of Transfusion Medicine, Mayo Clinic. Tn/mem cells were isolated using a three‐step procedure: first, CD14 and CD25 were depleted, and then CD62L was enriched using microbeads, following the manufacturer's protocol (Miltenyi Biotec, Bergisch Gladbach).

Blood samples from CLL patients with were obtained under a biorepository protocol approved by the Institutional Review Board of Mayo Clinic (20‐010888) and adhering to the ethical principles of the Declaration of Helsinki. Since this was a proof‐of‐concept study, patient selection criteria were broadly limited to individuals (male or female at ≥18 years of age) diagnosed with B‐cell lymphomas, CLL, or B‐cell ALL, who were willing to sign informed consent to provide additional blood or tissue during an already scheduled blood draw or biopsy. The patients were informed and provided written consent. The CAR T cells were produced from Pan T cells isolated with Pan T‐cell isolation kit (Miltenyi Biotec, Bergisch Gladbach), as previously described.^33^ The autologous B cells were isolated by using the EasySep Human B‐cell Isolation Kit (Stem Cell Technologies), following the protocol. The immunophenotyping of B cells included staining with anti‐BAFF‐R‐AF647 and anti‐CD19‐PE antibodies (BD Bioscience) for flow cytometry.

### 4.3 CAR T‐cell production

A second‐generation BAFF‐R‐CAR was developed, incorporating the humanized H90 BAFF‐R antibody scFv,^35^ CD4 transmembrane, 4‐1BB, and CD3ζ intracellular signaling domains, along with truncated EGFR serving as a marker and suicide switch.^34^ The pHIV.7 lentiviral vector were used for CAR clone as previous described.^34^ CD19‐CAR was created with the sample protocol using CD19 antibody scFv.^35^ Lentiviruses were produced and titered as previous described35. The Tn/mem T cells were isolated from healthy donor PBMCs and CAR T‐cell production were generated as previous described.^33^ In briefly, the Tn/mem cells were divided into two aliquots. One aliquot was used to create CAR T cells: these T cells were activated with Dynabeads Human T‐Activator CD3/CD28 (Thermo Fisher) for 24 h, transduced with CAR‐encoding lentivirus at a multiplicity of infection (MOI) of 1, then activated with CD3/CD28 beads for 6 days. After bead removal, CAR T cells were allowed to expand for an additional 7 days. The second aliquot was served without CAR lentivirus transduction as non‐CAR T, which were expanded using the same protocol but without CAR lentivirus infection. This CAR T‐cell production protocol can be easily translated to a GMP facility for generation of clinical grade cellular product; however, due to the significant numbers of T cells required for patient treatment, T cells would be isolated using leukapheresis for clinically produced CAR T cells. CLL patient derived CAR T cells were generated following the same protocol except using pan T cells due to the technical challenge of isolating sufficient Tn/mem cells from limited patient blood.

### 4.4 In vitro functional assays

#### 4.4.1 Degranulation assay

CD107a degranulation assay follows the protocols described previously.^33^, ^35^, ^47^, ^64^ In briefly, incubate CAR T cells with target cells with medium containing GolgiStop reagent and CD107a antibody (both from BD Biosciences) for 4−6 h. After incubation, the cells were stained with CD3, CD4, CD8, and EGFR (BD Biosciences). The samples were analyzed using flow cytometer (either the Attune or the Fortessa flow cytometer), with data processing done through FlowJo software. The negative controls of this assay were non‐CAR T cells derived from the same healthy donor or patients.

#### 4.4.2 Cytokine release assay

Following empirical optimization, CAR T cells and target cells were co‐cultured at an E:T ratio (4:1) for 72 h. Then, the supernatant was collected and analyzed for IFN‐γ using an enzyme‐linked immunosorbent assay.^35^

#### 4.4.3 Direct killing assay

To evaluate the cytolysis of tumor cells by CAR T cells, GFP‐positive target cells were co‐incubated with CAR T cells at an E:T ratio (20:1) for 24 h as previously described and empirically optimized.^33^ The percentile of alive GFP‐expression tumor cells was identified with Sytox Blue (Thermo Fisher) using the Attune flow cytometer for analysis. The percentages of alive GFP‐expression tumor cells were quantified using a gating strategy that first identified the live cells and then the GFP‐positive cells.

### 4.5 Statistical analysis

The Student's *t*‐test was performed by using Prism Version software (GraphPad), and data were represented as means ± SEM. When comparing non‐CAR T and CAR T groups, significance levels were presented as follows: ^*^ *p* < 0.05; ^**^ *p* < 0.01; ^***^ *p* < 0.001. A bar over a data set pair also indicates statistical comparison.

## AUTHOR CONTRIBUTIONS

Yaqing Qie and Yan Luo designed and performed experiments and analyzed data. Qing Shao, Rocio Rivera‐Valentin, Yaqing Qie, Tommy To, Shuhua Li, and Andrew Liu performed experiments and analyzed data. Farah Yassine, Hemant S. Murthy, and Mohamed A. Kharfan‐Dabaja organized subject selection and sample collection. Mohamed A. Kharfan‐Dabaja, Hemant S. Murthy, Roxana Dronca, and Hong Qin discussed the research direction. Martha E. Gadd drafted and generated final figures and participated in the final review with Mohamed A. Kharfan‐Dabaja, Hemant S. Murthy, Hong Qin, and Yan Luo. All authors have reviewed and endorsed the final manuscript.

## CONFLICT OF INTEREST STATEMENT

H. Qin has equity ownership with Pepromene Bio Inc. The remaining authors declare they have no conflicts of interest.

## ETHICS STATEMENT

Written consent was provided by any participant in this study. The collection of samples complied with local legislative and institutional guidelines. The Mayo Clinic Institutional Review Board approved studies involving patients (ID 20‐010888).

## Supporting information

Supporting Information

## Data Availability

Raw data that support the conclusions of this article can be obtained by requesting them from the corresponding author. Patient and/or healthy donor‐related information cannot be shared due to confidentiality.
