# Supplementary material for: Targeting chronic lymphocytic leukemia with B‐cell activating factor receptor CAR T cells
Source: MedComm (2020). 2024 Sep 2;5(9):e716. doi: 10.1002/mco2.716 (PMC11366826; doi:10.1002/mco2.716)
Supplement: Supplementary file 1 — Supporting Information [file MCO2-5-e716-s001.docx]

Supplementary Material

**Targeting chronic lymphocytic leukemia with BAFF-R CAR-T cells**

**Qie Y, Gadd ME, Shao Q, To T, *et al.***

# Supplementary Data

# Supplementary Figures and Tables

## Supplementary Tables

**Supplementary Table 1. Characterization assays of three batches of BAFF-R CAR-T cells**

|  | **Production 1** | | **Production 2** | | **Production 3** | |
| --- | --- | --- | --- | --- | --- | --- |
| **Sample** | Non-CAR T cells | BAFF-R CAR-T cells | Non-CAR T cells | BAFF-R CAR-T cells | Non-CAR T cells | BAFF-R CAR-T cells |
| **Fold Expansion** | 48.3 | 57.5 | 35.8 | 50.2 | 50.3 | 53.7 |
| **Viability ( ≥ 70% at day 14)** | 96.7 | 97.5 | 98.7 | 98.5 | 99 | 98 |
| **Identity ( ≥ 80%)** | 90.3 | 91 | 96.8 | 96.3 | 99.4 | 99.8 |
| **Potency (EGFR ≥ 10%)** | 0.88 | 49.7 | 0.19 | 66.6 | 0.42 | 59.3 |
| **WPRE (Copies/cell)** | 0.0 | 1.63 | 0.06 | 0.82 | 0.07 | 0.34 |
| **VSVG (Copies/50ng DNA)** | 0.0 | 0.4 | 0.60 | 0.55 | 0.33 | 0.35 |

**Abbreviations: EGFR =** Epidermal growth factor receptor ; WPRE = Woodchuck hepatitis virus posttranscriptional regulatory element ; VSVG = vesicular stomatitis virus G glycoprotein

**Supplementary Table 2. Characterization assays of three batches of BAFF-R CAR-T cells generated from healthy donors.**

|  | | | Healthy Donor 1  (CD107a %)* | Healthy Donor 2  (CD107a %) | Healthy Donor 3  (CD107a %) |
| --- | --- | --- | --- | --- | --- |
| CD8 T cells | Non CAR-T | Nalm-6 WT | 6.16 | 2.08 | 2.84 |
|  |  | BAFF-R KO Nalm-6 | 5.95 | 3.39 | 3.29 |
|  | BAFF-R CAR-T | Nalm-6 WT | 65.0 | 47.4 | 37.5 |
|  |  | BAFF-R KO Nalm-6 | 8.01 | 7.69 | 2.52 |
| CD4 T cells | Non CAR-T | Nalm-6 WT | 1.58 | 3.56 | 0.81 |
|  |  | BAFF-R KO Nalm-6 | 1.80 | 3.42 | 1.12 |
|  | BAFF-R CAR-T | Nalm-6 WT | 28.3 | 35.8 | 31.6 |
|  |  | BAFF-R KO Nalm-6 | 2.76 | 9.06 | 1.95 |

*Data shown in Figure 1B and 1C.

Abbreviations: WT = wild type; KO = Knock out

# Supplementary Table 3. Clinical characteristics of selected patients diagnosed with CLL

| **Patient number** | **Age**  **(years)** | **Gender** | **Year of diagnosis** | **Rai Staging at Diagnosis** | **N of previous lines of therapy** | **Disease status at the time of phlebotomy** |
| --- | --- | --- | --- | --- | --- | --- |
| 1 | 77 | M | 2016 | 4 | 0  (observation) | PD |
| 2 | 64 | M | 2018 | 1 | 2  (obinutuzumab / chlorambucil, ibrutinib) | SD |
| 3 | 54 | F | 2017 | 0 | 0  (observation) | SD |
| 4 | 77 | F | 2007 | N/A | N/A | SD |
| 5 | 75 | M | 2020 | 0 | 0  (observation) | N/A |
| 6 | 42 | M | 2020 | 0 | N/A | N/A |
| 7 | 80 | M | 2005 | 4 | ibrutinib + CA-4948 (IRAK-4 inhibitor)  (2 cycles to date) | N/A |
| 8 | 78 | M | 2013 | 0 | 0  (observation) | SD |

**Abbreviations: N:** number; **PD:** progressive disease; **SD:** stable disease; **N/A** = not available

##
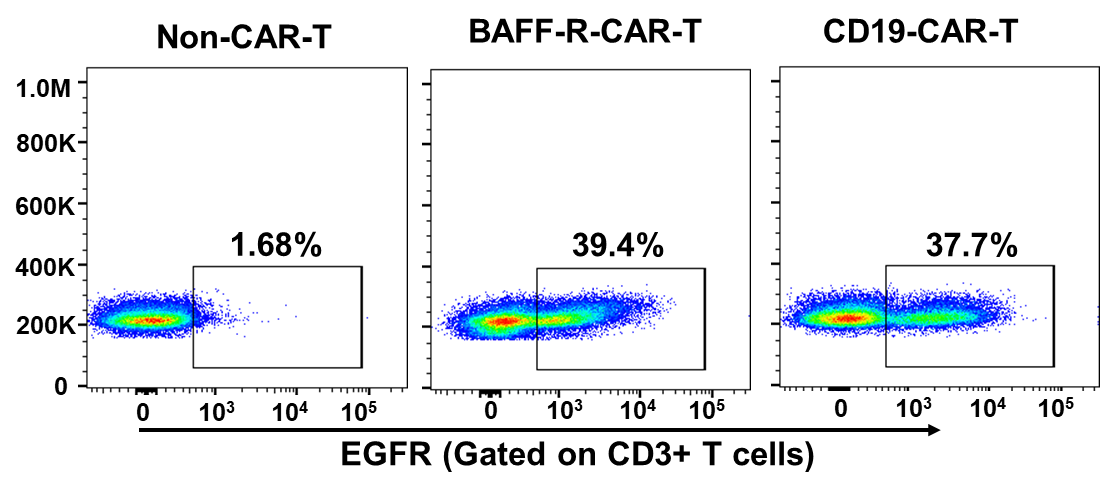
Supplementary Figures

**A**

**
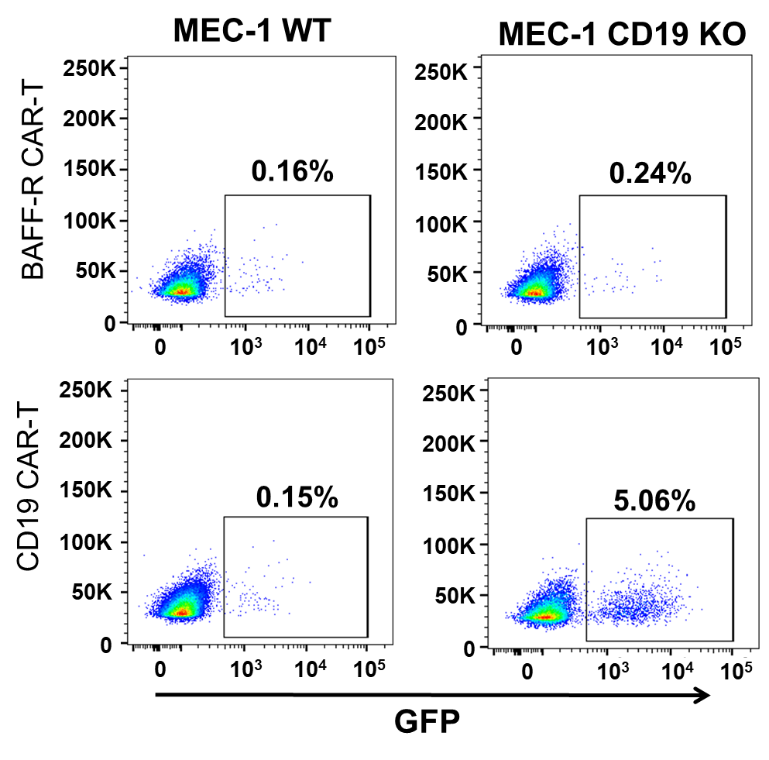
**

**B**

**Supplementary Figure 1.** **Characterization of CAR-T cells from healthy donor.** (A) Non-CAR-T, BAFF-R CAR-T cells, and CD19 CAR-T cells were generated from one healthy donor. The potency of the CAR-T cells was assessed using EGFR-antibody, gated on CD3 T cells. (B) Cytolysis of target cells were monitored in a direct killing assay in which target cells were labeled with GFP and incubated with either BAFF-R CAR-T cells or CD19 CAR-T cells. Target cells were either MEC-1 WT or MEC-1 CD19 KO cells to highlight the antigen specific cytotoxicity of the CAR-T cells.


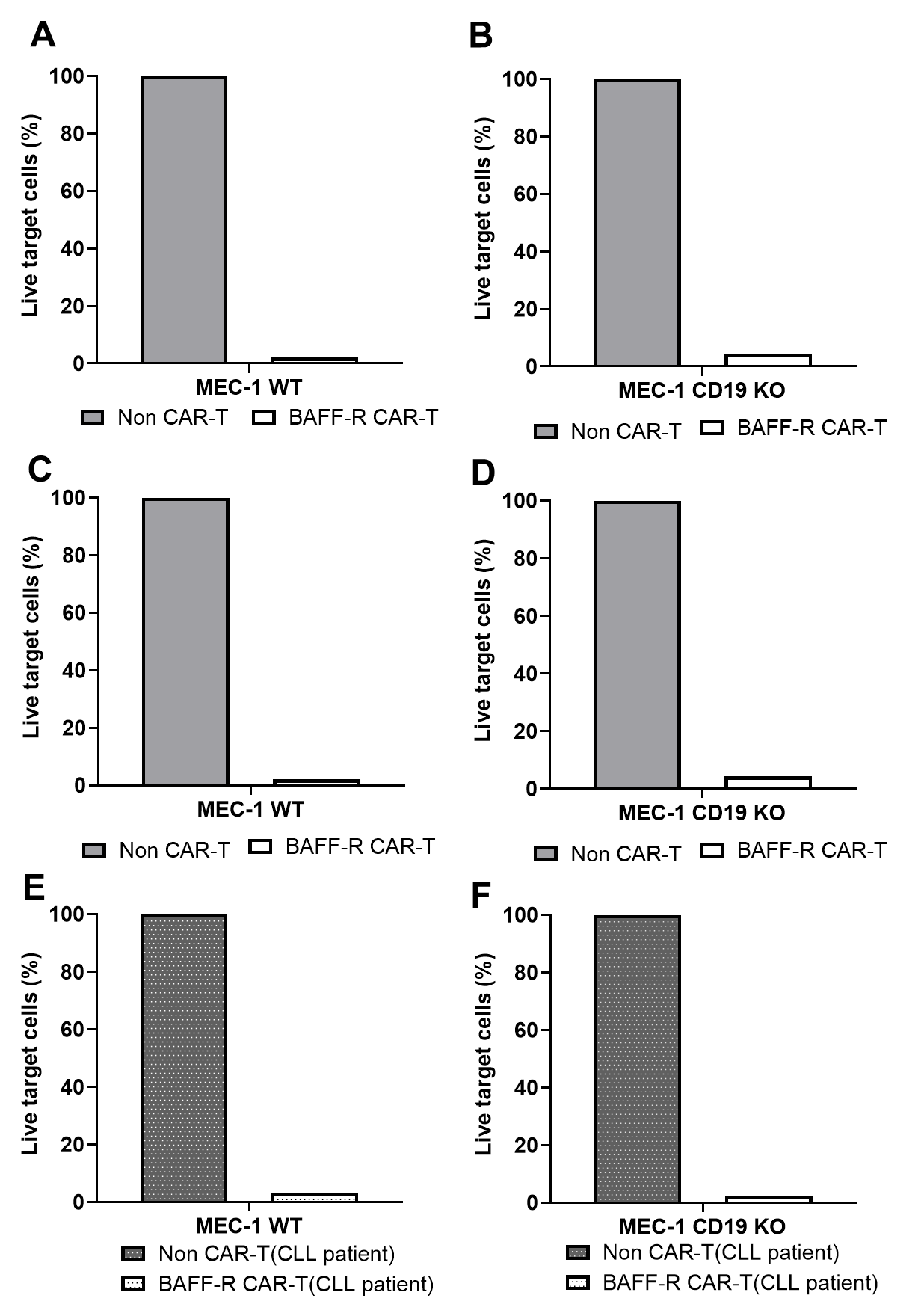


**Supplementary Figure 2. Direct cytotoxicity of BAFF-R CAR-T cells against chronic lymphocytic leukemia (CLL) cell lines.** Following the same experimental design as in Figure 2, the direct cytotoxicity of BAFF-R CAR-T cells (from healthy donors or CLL patient) against either MEC-1 WT or MEC-1 CD19 KO cells was assessed. The live GFP-labeled target cells in the Non-CAR-T group were utilized to normalize the percentage of remaining live target cells in BAFF-R CAR-T group from the same donor for the same target cells. BAFF-R CAR-T cells were engineered from healthy donor 1 (A, B) and healthy donor 2 (C, D); CLL Patient 2 was included as the representative CLL patient (E, F).

**
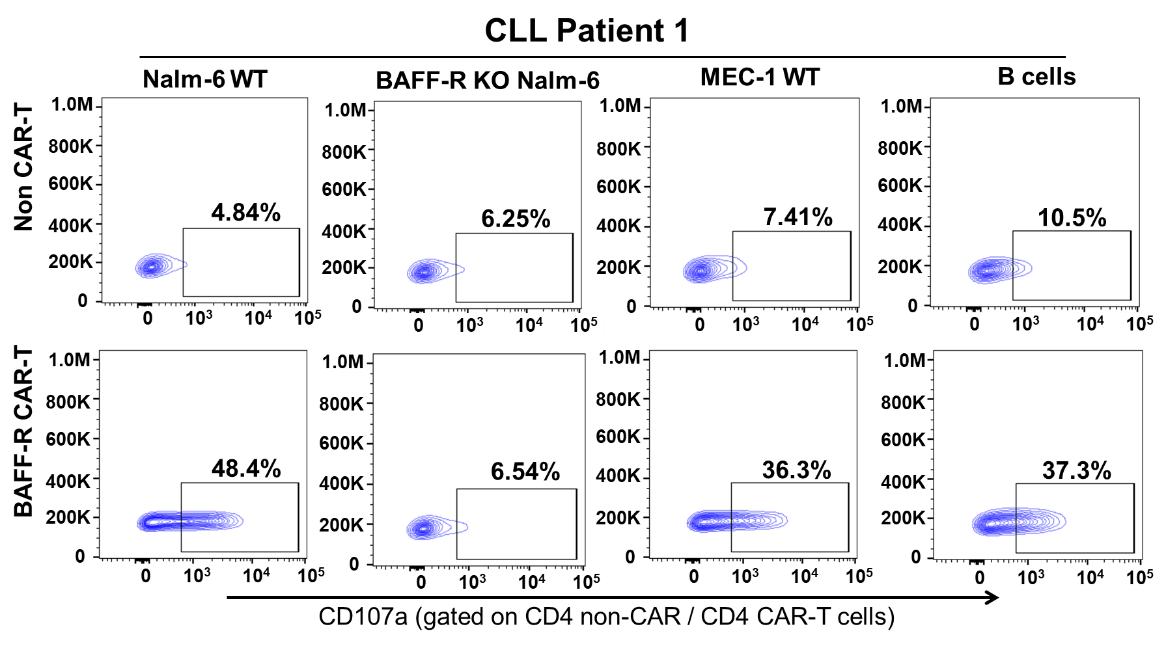
**

**A**

**
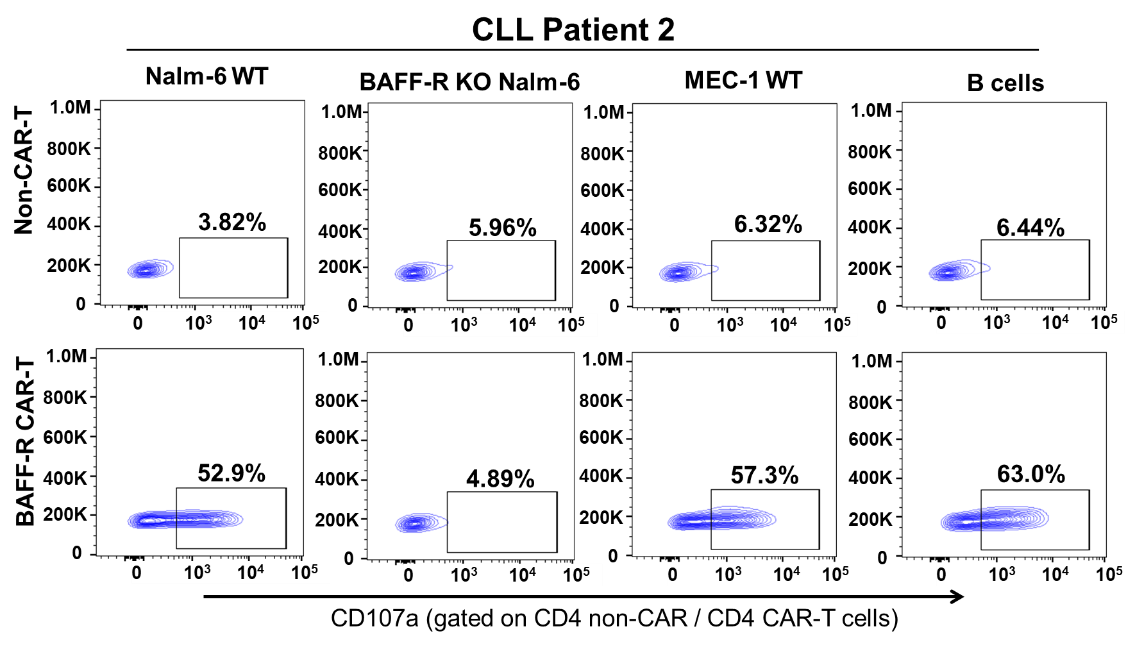
**

**B**

**
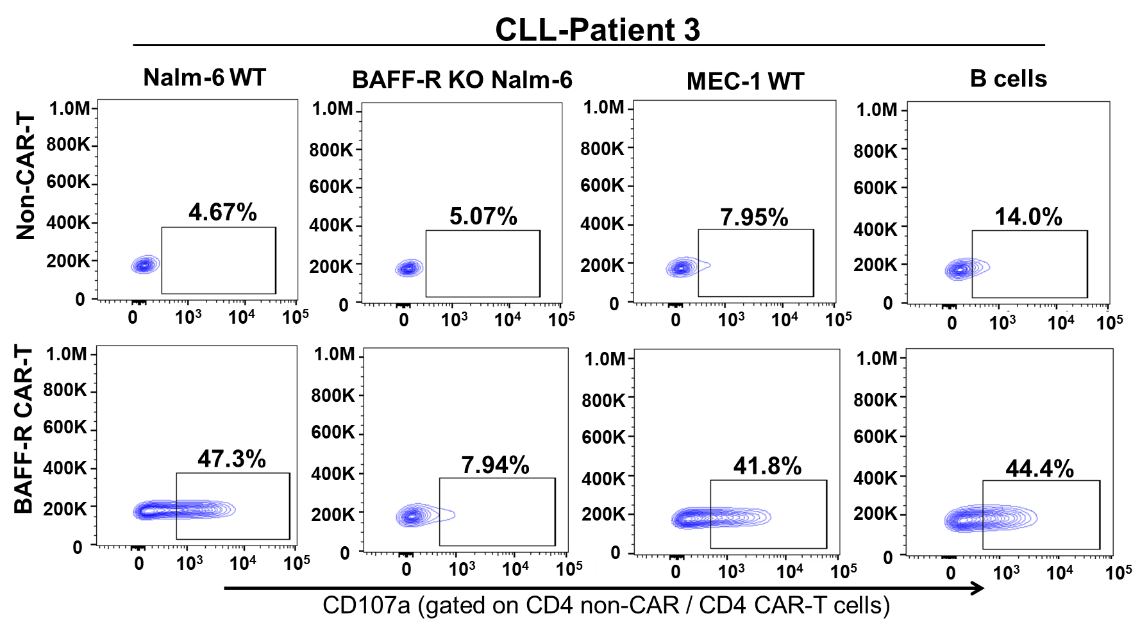
**

**C**

**Supplementary Figure 3. CLL patient-derived BAFF-R CAR-T cells elicited ex vivo cytotoxicity against autologous B cells.** Using a CD107a degranulation assay, cytotoxicity of CLL patient-derived BAFF-R CAR-T cells was evaluated against a collection of target cells. Nalm-6 WT, BAFF-R KO Nalm-6, and MEC-1 WT cells were employed as control target cells. Most significantly, cytotoxicity of CLL patient-derived BAFF-R CAR-T cells showed cytotoxicity against autologous B cells. Non-CAR-T cells from the same patient were served as a negative control. The analysis of cytotoxicity was gated on the CD4^+^ CAR-T cell populations. (**A**) CLL patient 1, (**B**) CLL patient 2, and (**C**) CLL patient 3.


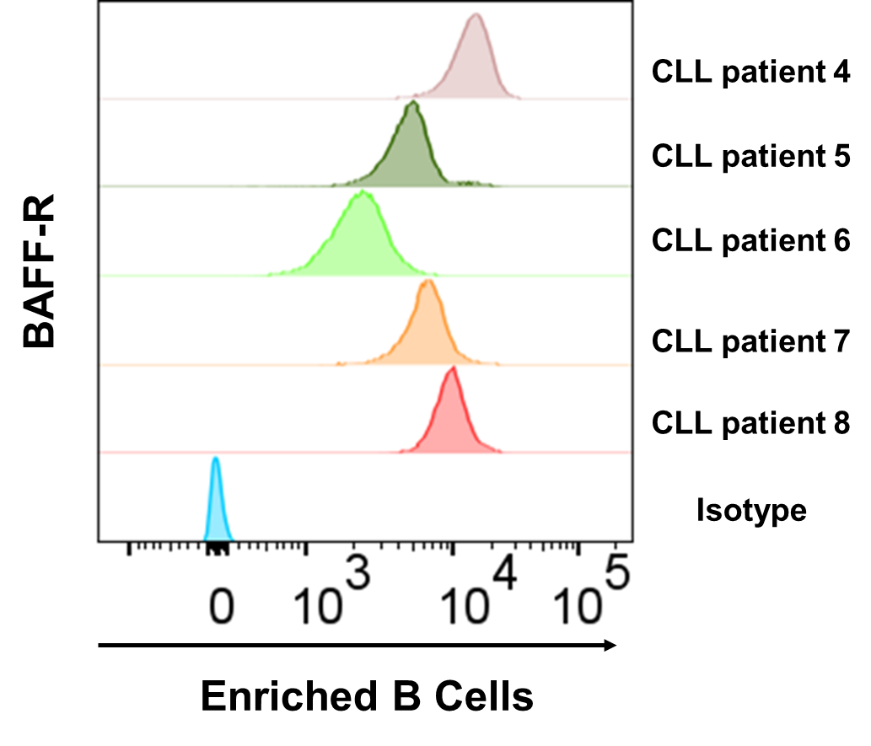


**Supplementary Figure 4. Evaluation of BAFF-R expression of CLL patient samples evaluated in house and reported in literature.** The expression of BAFF-R on the enriched B cells isolated from five CLL patients was characterized using the BAFF-R antibody in a flow cytometry analysis.
